# Supplementary figures and images for: Active degradation of MarA controls coordination of its downstream targets
Source: PLoS Comput Biol. 2018 Dec 27;14(12):e1006634. doi: 10.1371/journal.pcbi.1006634 (PMC6307708; doi:10.1371/journal.pcbi.1006634)

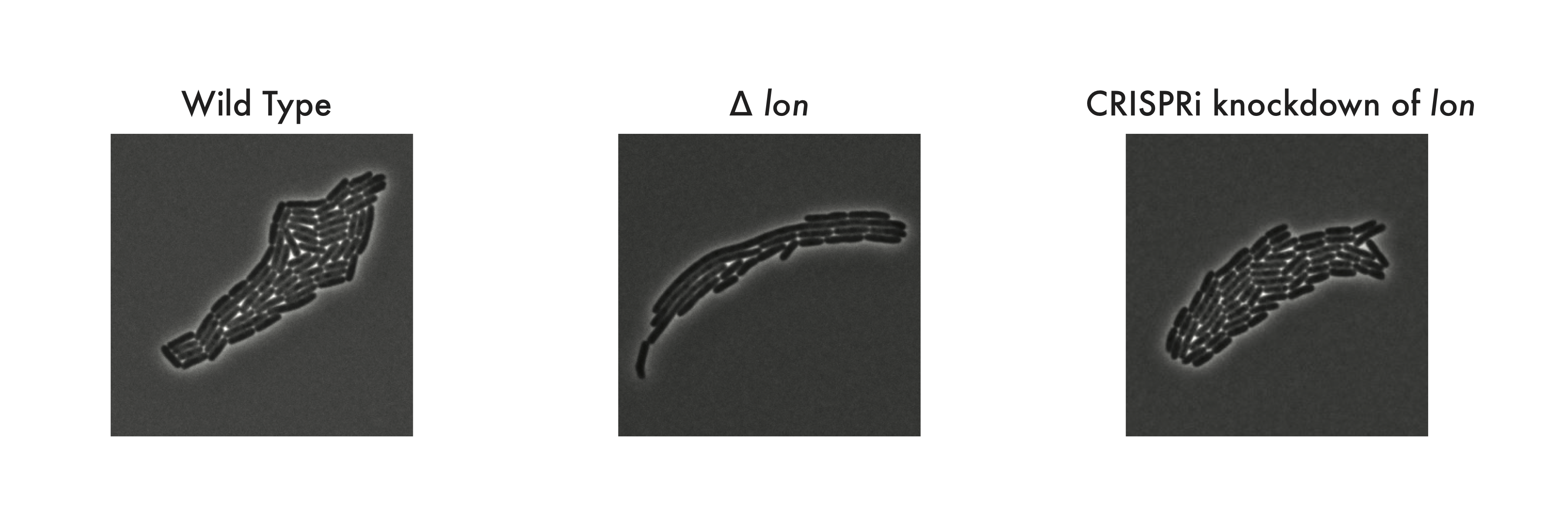

Supplement: S1 Fig — Phase contrast images of microcolonies after 200 minutes of growth. Slower growth rate and increased filamentation are evident in the Δlon strain. (TIFF) [file pcbi.1006634.s001.tiff]

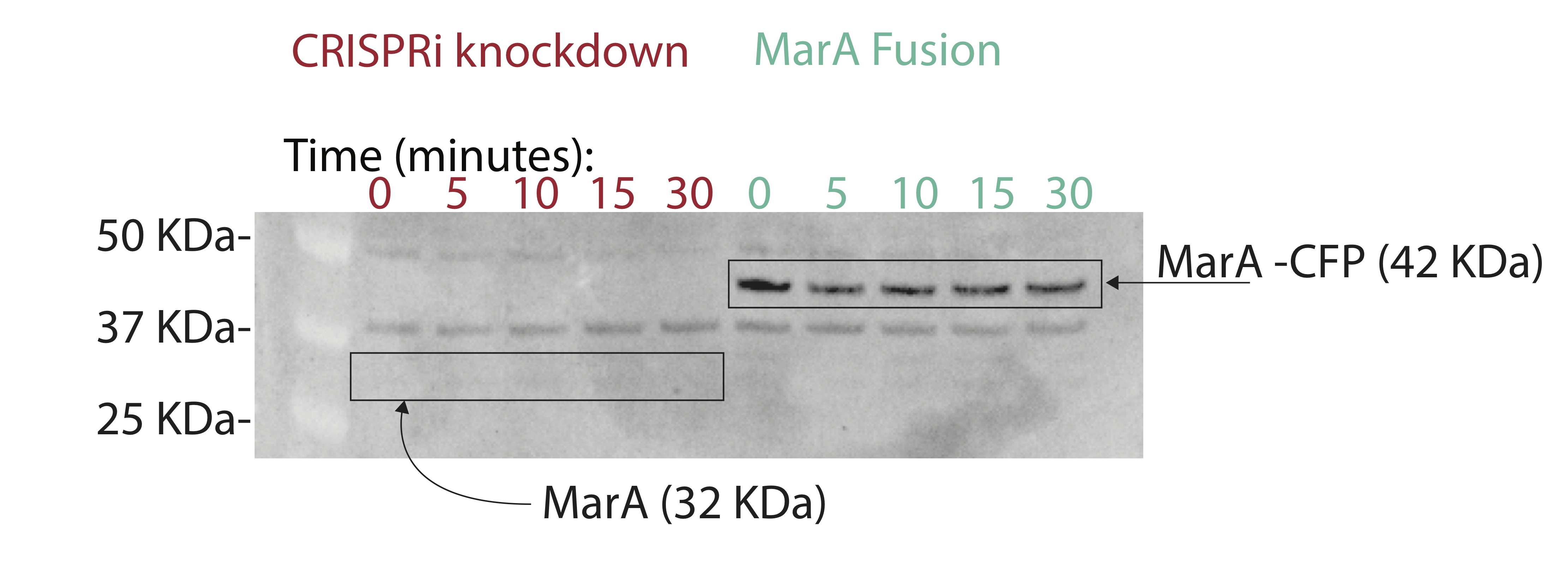

Supplement: S2 Fig — Minute markers represent when sample was taken after spectinomycin exposure. Missing bands in the expected 32 KDa range demonstrate that MarA does not exist in sufficient quantities even at t = 0 to be assayed via Western blot in the CRISPRi knockdown system due to rapid degradation. The band at 42 KDa is associated with the MarA-CFP fusion, which is stable and appears on the Western blot. For experimental details, see S1 File. (TIFF) [file pcbi.1006634.s002.tiff]

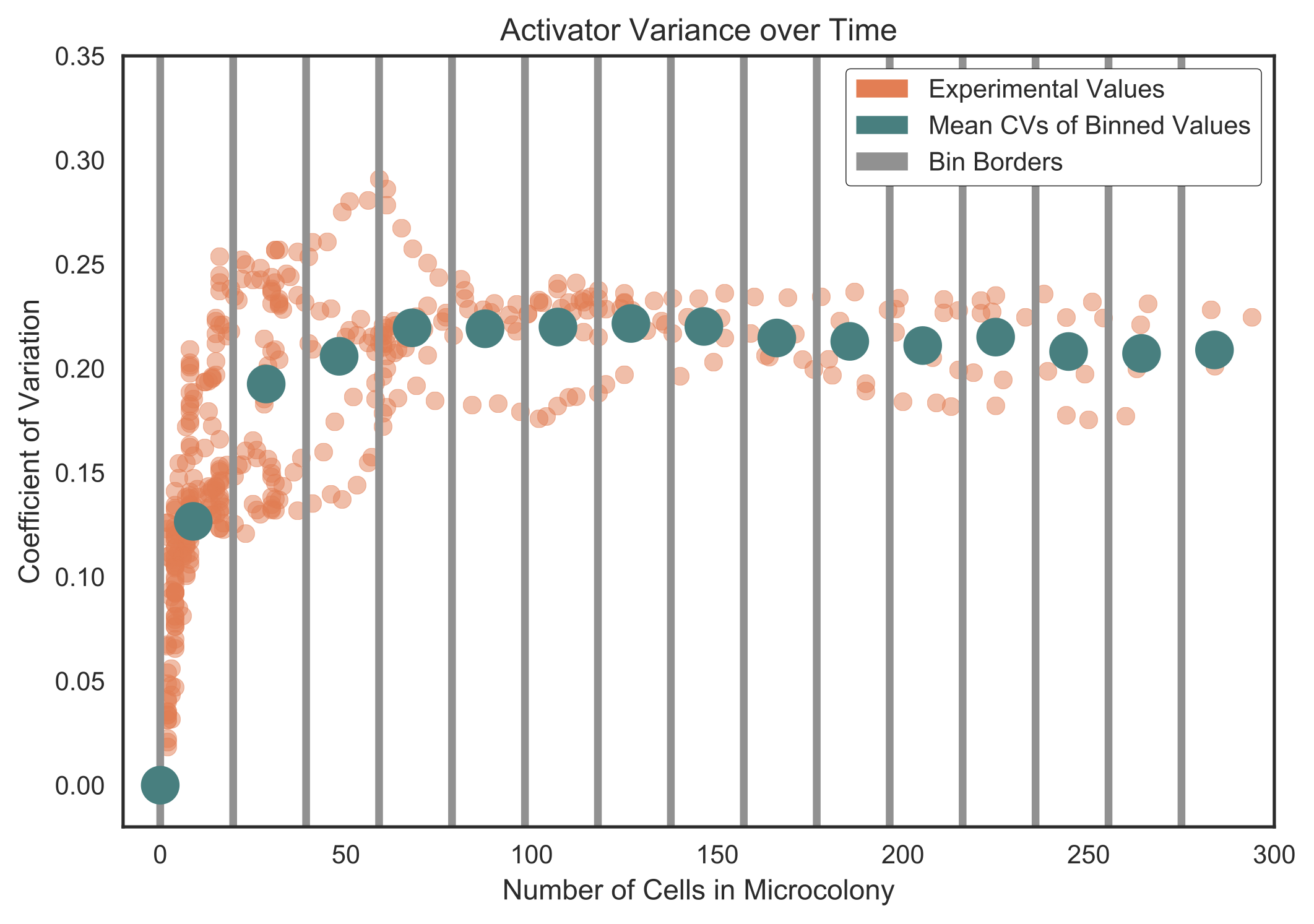

Supplement: S3 Fig — Microcolonies were allowed to grow for 300 minutes with data taken every 3 minutes. Variance was computed for each microcolony at each time point and then plotted against the number of cells in the microcolony (orange dots). These data were then binned (gray bars) and summary statistics such as the mean coefficient of variation across microcolonies (green dots) were generated for each bin. (TIFF) [file pcbi.1006634.s003.tiff]

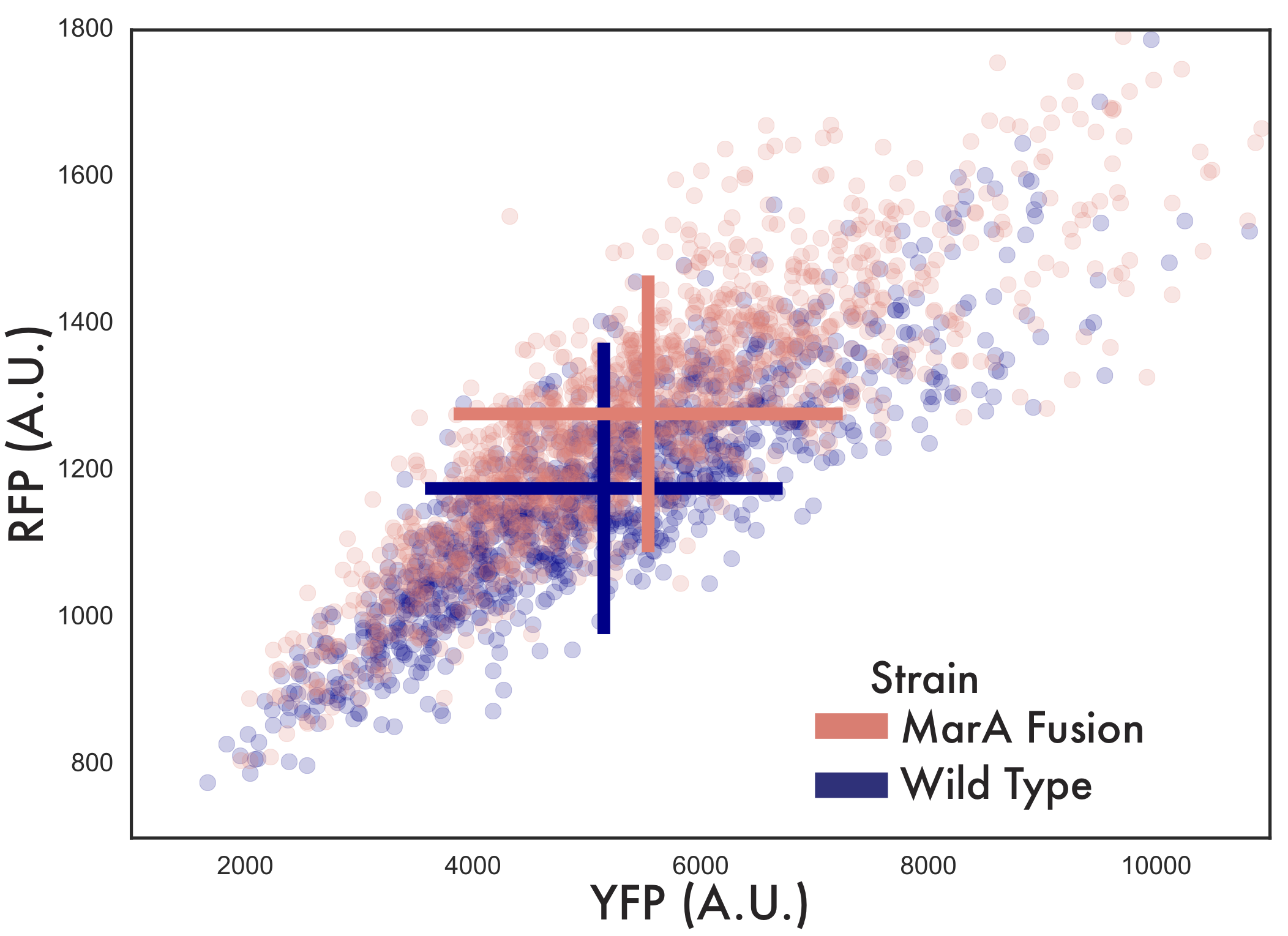

Supplement: S4 Fig — YFP (PinaA) and RFP (PacrAB) fluorescence values from growing microcolony data plotted without the time dimension. Error bars show means and standard deviations for the two bivariate distributions. 10 μM IPTG induced PlacUV5-MarA elicits a very similar downstream response to 0 μM IPTG induced PlacUV5-MarA-CFP translational fusion (two distributions are statistically equivalent by the 2D Kolmogorov-Smirnov test, p>0.1). (TIFF) [file pcbi.1006634.s004.tiff]

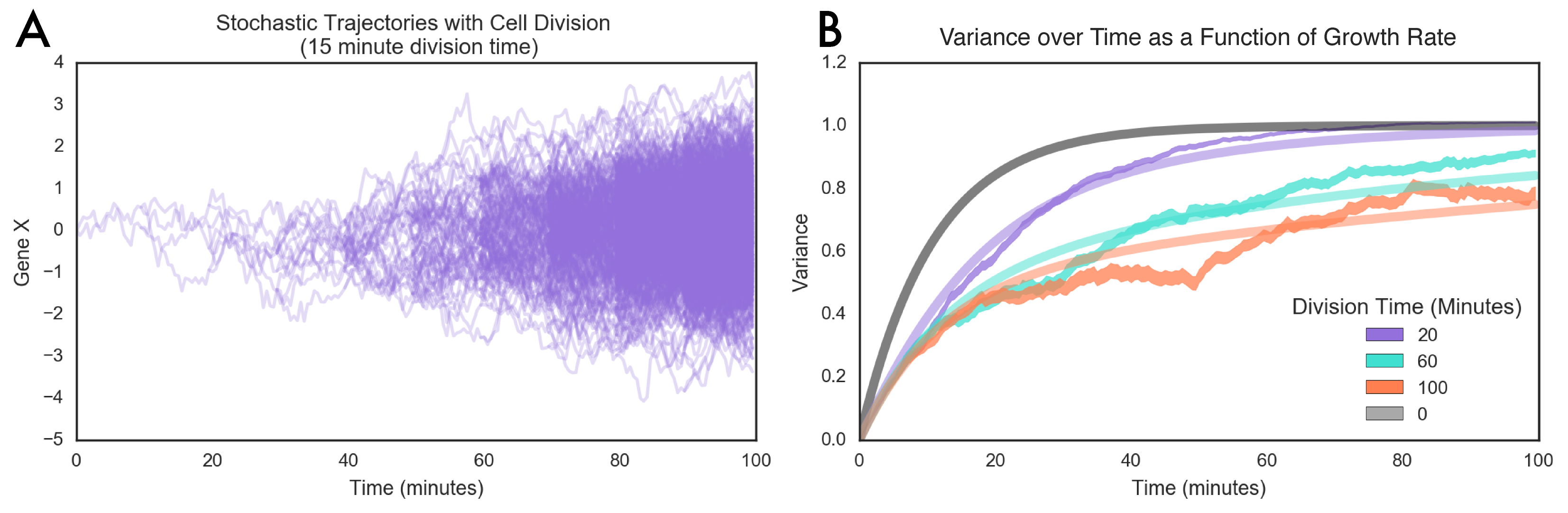

Supplement: S5 Fig — (A) Example simulation of a growing bacterial microcolony with a cell division time of 15 minutes. Individual trajectories show level of X. (B) Analytical solution to the variance functions including growth rate terms. Plot also shows the average variance for 1000 stochastic simulations with cell division. The shaded regions represent the standard error over all simulations centered around the mean for each simulation set. The solid lines represent the analytical solutions, with the gray line representing the theoretical maximum. See S1 File for growth rate functions. (TIF) [file pcbi.1006634.s005.tif]
