# Supplementary material for: Active degradation of MarA controls coordination of its downstream targets
Source: PLoS Comput Biol. 2018 Dec 27;14(12):e1006634. doi: 10.1371/journal.pcbi.1006634 (PMC6307708; doi:10.1371/journal.pcbi.1006634)
Supplement: S1 File — (PDF) [file pcbi.1006634.s009.pdf]

Supplementary Information

Active degradation of MarA controls coordination of its downstream targets
N. A. Rossi, T. Mora, A. M. Walczak, M. J. Dunlop

Plasmids

Reporters

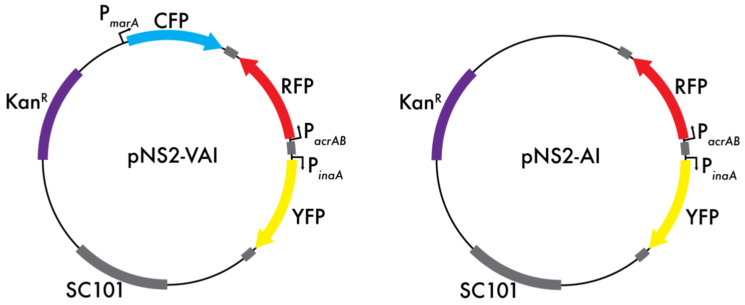

Plasmid maps of pNS2-VAI and pNS2-AI. These are the reporter plasmids for the MarA fusion and CRISPRi systems. pNS2-VAI includes three fluorescent protein genes (cerulean CFP, mCherry RFP, and venus YFP) under the control of the promoters as indicated, a low-copy SC101 origin of replication, and a kanamycin resistance marker. pNS2-AI is identical in terms of features with the absence of *cfp* and the corresponding promoter.

Construct	Primers
P_{acrAB}	F: GCCAGTAGATTGCACCGCG<br>R: TCGTGCTATGGTACATACATTCACA
P_{inaA}	F: CAATGCTTTTCAGCGTTAACTCTG<br>R: ACGACAATGACTATAGGTGGT
P_{marA}	F: GGTTGTTATCCTGTGTATCTGG<br>R: ATTAGTTGCCCTGGCAAG

Table 1: Primers for construction of transcriptional reporter plasmids

CRISPRi knock down

To knock down endogenous Lon protease we used the plasmids and methods from [1]. To target the *lon* gene, we designed the sequence TAAACCACCACATT̲GCGCAG which binds ~ 100

base-pairs after the transcriptional start site of the *lon* gene. This sequence has a single mutation (underlined letter) at the 14th base-pair. This decreases its repression and causes leaky expression of *lon* to avoid producing off-target phenotypic changes such as increased filamentation (Fig. S1).

lon knockout

To knock out *lon* for Fig. S1 we used the primers from [2]: TCGTGTCATCTGATTACCTGGCG-GAAATTAAACTAAGAGAGAGCTCTATGATTCCGGGGATCCGTCGACC and TTTTTATTA GTGCATTTTGCGCGAGGTCACTATTTTGCAGTCACAACCTGTGTAGGCTGGAGCTGC TTCG along with the pKD13 plasmid [3].

Western Blots

To determine the half-life of both wild type MarA under CRISPRi knockdown of Lon protease and MarA fusion protein, we grew cultures of both systems overnight and then diluted them 1:100 in 30 ml LB. Cells were grown for 4 hours and then spectinomycin (100 μg/ml) was added to stop protein synthesis. 1 ml of the culture was collected at different time points (0, 5, 10, 15 and 30 minutes). Cells were harvested at 13,000 rpm for 3 minutes and the supernatant was removed in order to extract total proteins. The cell pellets were resuspended with 4$\%$ SDS and shaken for 15 minutes at 37°C. Glass beads (0.1 mm) were added and the samples were vortexed for 10 seconds and incubated on ice for 10 seconds three times. Anti-MarA antibody was used to detect the presence of MarA or MarA fusion protein. Membranes were probed first with 1:3000 of rabbit anti-MarA antibodies generously provided by Valerie Duval, Laura McMurry, and Stuart Levy [4]. Primary antibodies were detected using the secondary Peroxidase-AffiniPure Goat Anti-Rabbit IgG.

Analytic solutions for variance and mutual information over time

Following the methods outlined in [5] we derived the exact analytical solutions for the models described in the text. This allowed us to describe how variance evolves as a function of time for the input X and its two downstream targets Y and Z.

$$Var\{x(t)\} = \frac{1}{2}\tau_x\left(1-e^{-\frac{2t}{\tau_x}}\right) \tag{1}$$

$$\begin{aligned} Var\{y(t)\} = \frac{\tau_y e^{-2t\left(\frac{1}{\tau_y}+\frac{1}{\tau_x}\right)}}{2\left(\tau_x-\tau_y\right)^2\left(\tau_x+\tau_y\right)} & \left(\frac{g_y^2\tau_x^4}{\tau_y}e^{\frac{2t}{\tau_y}}\left(e^{\frac{2t}{\tau_x}}-1\right)\right. \\ & + \tau_x^3\left(e^{\frac{2t}{\tau_x}}\left(e^{\frac{2t}{\tau_y}}-1\right)-g_y^2\left(e^{\frac{2t}{\tau_x}}-4e^{t\left(\frac{1}{\tau_y}+\frac{1}{\tau_x}\right)}+2e^{2t\left(\frac{1}{\tau_y}+\frac{1}{\tau_x}\right)}+e^{\frac{2t}{\tau_y}}\right)\right) \\ & \left. + \tau_y\tau_x^2\left(g_y^2-1\right)e^{\frac{2t}{\tau_x}}\left(e^{\frac{2t}{\tau_y}}-1\right)-\tau_y^2\tau_x e^{\frac{2t}{\tau_x}}\left(e^{\frac{2t}{\tau_y}}-1\right)+\tau_y^3 e^{\frac{2t}{\tau_x}}\left(e^{\frac{2t}{\tau_y}}-1\right)\right) \end{aligned} \tag{2}$$

$$
\begin{aligned}
Var\{z(t)\} = \frac{\tau_z e^{-2t\left(\frac{1}{\tau_z}+\frac{1}{\tau_x}\right)}}{2\left(\tau_x-\tau_z\right)^2\left(\tau_x+\tau_z\right)} \Bigg(& \frac{g_z^2\tau_x^4}{\tau_z} e^{\frac{2t}{\tau_z}}\left(e^{\frac{2t}{\tau_x}}-1\right) \\
& + \tau_x^3\left(e^{\frac{2t}{\tau_x}}\left(e^{\frac{2t}{\tau_z}}-1\right) - g_z^2\left(e^{\frac{2t}{\tau_x}} - 4e^{t\left(\frac{1}{\tau_z}+\frac{1}{\tau_x}\right)} + 2e^{2t\left(\frac{1}{\tau_z}+\frac{1}{\tau_x}\right)} + e^{\frac{2t}{\tau_z}}\right)\right) \\
& + \tau_z\tau_x^2\left(g_z^2-1\right)e^{\frac{2t}{\tau_x}}\left(e^{\frac{2t}{\tau_z}}-1\right) - \tau_z^2\tau_x e^{\frac{2t}{\tau_x}}\left(e^{\frac{2t}{\tau_z}}-1\right) + \tau_z^3 e^{\frac{2t}{\tau_x}}\left(e^{\frac{2t}{\tau_z}}-1\right)\Bigg)
\end{aligned} \tag{3}
$$

Next, in order to quantify how coordinated diversity of two downstream genes evolves over time we compute the mutual information between the two genes as a function of time. To compute this, we first calculate the covariance over time.

$$
\begin{aligned}
Cov\{y(t), z(t)\} = & \frac{\tau_x^2\tau_y\tau_z}{2\left(\tau_x^2-\tau_y^2\right)\left(\tau_y+\tau_z\right)\left(\tau_x^2-\tau_z^2\right)\left(\tau_x\left(\tau_y+\tau_z\right)-2\tau_y\tau_z\right)} \Bigg(\frac{g_y^2}{\tau_y}(\tau_x \\
& + \tau_z)\, e^{-t\left(\frac{2}{\tau_y}+\frac{1}{\tau_z}+\frac{3}{\tau_x}\right)}\Bigg(\left(\tau_y+\tau_z\right)\tau_x^3 e^{t\left(\frac{2}{\tau_y}+\frac{1}{\tau_z}+\frac{1}{\tau_x}\right)}\left(e^{\frac{2t}{\tau_x}}-1\right) \\
& + \tau_x^2 e^{t\left(\frac{1}{\tau_y}+\frac{1}{\tau_x}\right)}\Bigg(2\tau_y^2 e^{t\left(\frac{1}{\tau_z}+\frac{1}{\tau_x}\right)} - \tau_y^2 e^{t\left(\frac{1}{\tau_z}+\frac{1}{\tau_y}\right)} + 4\tau_z\tau_y e^{t\left(\frac{1}{\tau_z}+\frac{1}{\tau_x}\right)} - 2\tau_z^2 e^{\frac{2t}{\tau_x}} + 2\tau_z^2 e^{t\left(\frac{1}{\tau_z}+\frac{1}{\tau_x}\right)} + \\
& \tau_z^2 e^{t\left(\frac{1}{\tau_z}+\frac{1}{\tau_y}\right)} - \left(\tau_y^2+4\tau_z\tau_y+\tau_z^2\right)e^{t\left(\frac{1}{\tau_y}+\frac{1}{\tau_z}+\frac{2}{\tau_x}\right)}\Bigg) \\
& + \tau_y\tau_z\left(\tau_y+\tau_z\right)\tau_x e^{t\left(\frac{1}{\tau_y}+\frac{1}{\tau_z}+\frac{1}{\tau_x}\right)}\left(-4e^{\frac{t}{\tau_x}} + 3e^{t\left(\frac{1}{\tau_y}+\frac{2}{\tau_x}\right)} + e^{\frac{t}{\tau_y}}\right) \\
& - 2\tau_y^2\tau_z^2 e^{t\left(\frac{1}{\tau_y}+\frac{3}{\tau_x}\right)}\left(e^{t\left(\frac{1}{\tau_z}+\frac{1}{\tau_y}\right)}-1\right)\Bigg) + \frac{g_y^2}{\tau_y^2}(\tau_x \\
& + \tau_y)\,\tau_z e^{-t\left(\frac{1}{\tau_y}+\frac{2}{\tau_z}+\frac{3}{\tau_x}\right)}\Bigg(e^{t\left(\frac{1}{\tau_z}+\frac{1}{\tau_y}\right)}\Bigg(2\tau_x\left(\tau_y+\tau_z\right)\left(\tau_x\left(\tau_y+\tau_z\right)-2\tau_y\tau_z\right)e^{\frac{2t}{\tau_x}} + 2\left(\tau_x-\tau_y\right)e^{t\left(\frac{1}{\tau_z}+\frac{2}{\tau_x}\right)} \\
& \left(\left(\left(\tau_y+\tau_z\right)\tau_x^2 - \tau_y\tau_z\tau_x + \tau_y\tau_z^2\right)\sinh\left(\frac{t}{\tau_x}\right) + \tau_z\left(\tau_y\tau_z - \tau_x\left(2\tau_y+\tau_z\right)\right)\cosh\left(\frac{t}{\tau_x}\right)\right)\Bigg) \\
& - 2\tau_y^2\left(\tau_x^2-\tau_z^2\right)e^{t\left(\frac{1}{\tau_z}+\frac{3}{\tau_x}\right)}\Bigg)\Bigg)
\end{aligned} \tag{4}
$$

Next, the correlation of Y and Z is calculated by combining equations 2, 3, and 4 as :

$$
Corr\{y(t), z(t)\} = \frac{Cov\{y(t), z(t)\}^2}{Var\{y(t)\}Var\{z(t)\}} \tag{5}
$$

This is then converted to mutual information by the following equivalence

$$
I\{y(t), z(t)\} = -\frac{1}{2} ln(1 - Corr\{y(t), z(t)\}^2) \tag{6}
$$

Note this analytical conversion assumes Gaussian statistics for the underlying stochastic differential equations.

Analytic solutions for variance and mutual information over time with scaled variance

The solutions to the equations as illustrated in Figure 3 in the main text are similar to that in Figure 2 except the variance is constrained as a function of correlation time τ.

$$Var\{x(t)\} = 1 - e^{-\frac{2t}{\tau_x}} \tag{7}$$

$$\begin{aligned} Var\{y(t)\} = \frac{e^{-t\left(\frac{1}{\tau_x}+\frac{1}{\tau_y}\right)}}{(\tau_x-\tau_y)^2(\tau_x+\tau_y)} \Big(& -g_y^2\tau_x^2(\tau_x+\tau_y)\, e^{t\left(\frac{1}{\tau_y}-\frac{1}{\tau_x}\right)} \\ & + (\tau_x-\tau_y)^2\left(g_y^2\tau_x+\tau_x+\tau_y\right) e^{t\left(\frac{1}{\tau_x}+\frac{1}{\tau_y}\right)} \\ & - (\tau_x+\tau_y)\left(\tau_x\tau_y\left(g_y^2-2\right)+\tau_x^2+\tau_y^2\right) e^{t\left(\frac{1}{\tau_x}-\frac{1}{\tau_y}\right)} + 4g_y^2\tau_x^2\tau_y \Big) \end{aligned} \tag{8}$$

$$\begin{aligned} Var\{z(t)\} = \frac{e^{-t\left(\frac{1}{\tau_x}+\frac{1}{\tau_z}\right)}}{(\tau_x-\tau_z)^2(\tau_x+\tau_z)} \Big(& -g_z^2\tau_x^2(\tau_x+\tau_z)\, e^{t\left(\frac{1}{\tau_z}-\frac{1}{\tau_x}\right)} \\ & + (\tau_x-\tau_z)^2\left(g_z^2\tau_x+\tau_x+\tau_z\right) e^{t\left(\frac{1}{\tau_x}+\frac{1}{\tau_z}\right)} \\ & - (\tau_x+\tau_z)\left(\tau_x\tau_z\left(g_z^2-2\right)+\tau_x^2+\tau_z^2\right) e^{t\left(\frac{1}{\tau_x}-\frac{1}{\tau_z}\right)} + 4g_z^2\tau_x^2\tau_z \Big) \end{aligned} \tag{9}$$

$$\begin{aligned} Cov\{y(t), z(t)\} = & \frac{e^{-t\left(\frac{1}{\tau_y}+\frac{1}{\tau_z}+\frac{3}{\tau_x}\right)}}{\left(\tau_x^2-\tau_y^2\right)(\tau_y+\tau_z)\left(\tau_x^2-\tau_z^2\right)\left(\tau_x(\tau_y+\tau_z)-2\tau_y\tau_z\right)}\tau_x\tau_y\tau_z \Bigg(\tau_y(\tau_x \\ & + \tau_z)\Bigg(e^{t\left(\frac{1}{\tau_y}+\frac{1}{\tau_z}+\frac{1}{\tau_x}\right)}\left(-1+e^{\frac{2t}{\tau_x}}\right)(\tau_y+\tau_z)\tau_x^3 \\ & + \Bigg(2e^{t\left(\frac{1}{\tau_z}+\frac{2}{\tau_x}\right)}\tau_y^2 - e^{t\left(\frac{1}{\tau_y}+\frac{1}{\tau_z}+\frac{1}{\tau_x}\right)}\tau_y^2 + 4e^{t\left(\frac{1}{\tau_z}+\frac{2}{\tau_x}\right)}\tau_z\tau_y - 2e^{\frac{3t}{\tau_x}}\tau_z^2 + 2e^{t\left(\frac{1}{\tau_z}+\frac{2}{\tau_x}\right)}\tau_z^2 + e^{t\left(\frac{1}{\tau_y}+\frac{1}{\tau_z}+\frac{1}{\tau_x}\right)}\tau_z^2 - \\ & e^{t\left(\frac{1}{\tau_y}+\frac{1}{\tau_z}+\frac{3}{\tau_x}\right)}\left(\tau_y^2+4\tau_z\tau_y+\tau_z^2\right)\Bigg)\tau_x^2 \\ & + e^{t\left(\frac{1}{\tau_z}+\frac{1}{\tau_x}\right)}\left(-4e^{\frac{t}{\tau_x}}+3e^{t\left(\frac{1}{\tau_y}+\frac{2}{\tau_x}\right)}+e^{\frac{t}{\tau_y}}\right)\tau_y\tau_z(\tau_y+\tau_z)\tau_x \\ & - 2e^{\frac{3t}{\tau_x}}\left(-1+e^{t\left(\frac{1}{\tau_z}+\frac{1}{\tau_y}\right)}\right)\tau_y^2\tau_z^2\Bigg)\frac{g_y^2}{\tau_y^2} \\ & + \frac{g_z^2}{\tau_z^2}(\tau_x+\tau_y)\tau_z\Bigg(e^{t\left(\frac{1}{\tau_y}+\frac{1}{\tau_z}+\frac{1}{\tau_x}\right)}\left(-1+e^{\frac{2t}{\tau_x}}\right)(\tau_y+\tau_z)\tau_x^3 \\ & + \Bigg(-2e^{\frac{3t}{\tau_x}}\tau_y^2 + 2e^{t\left(\frac{1}{\tau_y}+\frac{2}{\tau_x}\right)}\tau_y^2 + e^{t\left(\frac{1}{\tau_y}+\frac{1}{\tau_z}+\frac{1}{\tau_x}\right)}\tau_y^2 + 4e^{t\left(\frac{1}{\tau_y}+\frac{2}{\tau_x}\right)}\tau_z\tau_y + 2e^{t\left(\frac{1}{\tau_y}+\frac{2}{\tau_x}\right)}\tau_z^2 - e^{t\left(\frac{1}{\tau_y}+\frac{1}{\tau_z}+\frac{1}{\tau_x}\right)}\tau_z^2 - \\ & e^{t\left(\frac{1}{\tau_y}+\frac{1}{\tau_z}+\frac{3}{\tau_x}\right)}\left(\tau_y^2+4\tau_z\tau_y+\tau_z^2\right)\Bigg)\tau_x^2 + e^{t\left(\frac{1}{\tau_y}+\frac{1}{\tau_x}\right)}\left(-4e^{\frac{t}{\tau_x}}+3e^{t\left(\frac{1}{\tau_z}+\frac{2}{\tau_x}\right)}+e^{\frac{t}{\tau_z}}\right)\tau_y\tau_z(\tau_y+\tau_z)\tau_x \\ & - 2e^{\frac{3t}{\tau_x}}\left(-1+e^{t\left(\frac{1}{\tau_z}+\frac{1}{\tau_y}\right)}\right)\tau_y^2\tau_z^2\Bigg)\Bigg) \end{aligned} \tag{10}$$

Modified model incorporating growth rate

To consider the effects of growth rate on the system we modified Eqn. 7 to include terms modeling the exponential growth of a bacterial microcolony.

$$Var\{x(t)\} = \left(1 - e^{-\frac{2t}{\tau_x}}\right)\left(1 - \frac{1}{N_{cells}e^{\frac{ln(2)}{t_{div}}t}}\right) \qquad (11)$$

where N_{cells} is the starting number of cells, t_{div} is the division time (or length of cell cycle) in minutes. τ_x is the correlation time of the activator, while t is measured in minutes.

Parameters

Parameter	Value	Definition
λ_x	range from 1 to 100	half-life of x
τ_x	$\frac{\lambda_x}{log(2)}$	correlation-time of x
τ_y	$\frac{30.0}{log(2)}$	correlation-time of y
τ_z	$\frac{30.0}{log(2)}$	correlation-time of z
g_y	$0.1\tau_y$	dose response gain of x on y
g_z	$0.1\tau_z$	dose response gain of x on z

Table 2: Parameters used for stochastic simulations and analytical solutions

The correlation time of y and z was assumed to scale from the average division time of *E. coli* as 30 minutes [6] as they are both stable proteins. The gain of each promoter was an approximation of *acrAB* and *inaA* promoters at wild-type levels of MarA [7].

References

1. Lei S Qi, Matthew H Larson, Luke A Gilbert, Jennifer A Doudna, Jonathan S Weissman, Adam P Arkin, and Wendell A Lim. Repurposing crispr as an rna-guided platform for sequence-specific control of gene expression. *Cell*, 152(5):1173–1183, 2013.

2. Tomoya Baba, Takeshi Ara, Miki Hasegawa, Yuki Takai, Yoshiko Okumura, Miki Baba, Kirill A Datsenko, Masaru Tomita, Barry L Wanner, and Hirotada Mori. Construction of escherichia coli k-12 in-frame, single-gene knockout mutants: the keio collection. *Molecular systems biology*, 2(1), 2006.

3. Kirill A Datsenko and Barry L Wanner. One-step inactivation of chromosomal genes in escherichia coli k-12 using pcr products. *Proceedings of the National Academy of Sciences*, 97(12):6640–6645, 2000.

4. Patrick F McDermott, David G White, Isabelle Podglajen, Michael N Alekshun, and Stuart B Levy. Multidrug resistance following expression of the escherichia coli mara gene in mycobacterium smegmatis. *Journal of bacteriology*, 180(11):2995–2998, 1998.

5. Daniel T Gillespie. Exact numerical simulation of the ornstein-uhlenbeck process and its integral. *Physical review E*, 54(2):2084, 1996.

6. D Joseph Clark and ODNA Maaløe. Dna replication and the division cycle in escherichia coli. *Journal of Molecular Biology*, 23(1):99–112, 1967.

7. Nicholas A Rossi and Mary J Dunlop. Customized regulation of diverse stress response genes by the multiple antibiotic resistance activator mara. *PLoS computational biology*, 13(1):e1005310, 2017.
